# Gender-Specific Association between Serum Uric Acid and Incident Fundus Arteriosclerosis in Chinese Population: A Retrospective Cross-Sectional Study

**DOI:** 10.1038/s41598-020-65575-z

**Published:** 2020-05-25

**Authors:** Qianqian Liu, Chunxing Liu, Yonghui Gao, Xinyan Zhang, Nengjun Yi, Jianping Cao, Yamin Wang, Yongbin Jiang, Zaixiang Tang

**Affiliations:** 1 Department of Biostatistics, School of Public Health, Medical College of Soochow University, Suzhou, 215123 China; 2 Jiangsu Key Laboratory of Preventive and Translational Medicine for Geriatric Diseases, Medical College of Soochow University, Suzhou, 215123 China; 3 Department of Laboratory, Hua Dong Sanatorium, Wuxi, 214065 China; 4 Department of Biostatistics, Jiann-Ping Hsu College of Public Health, Georgia Southern University, Statesboro, GA 30458 USA; 5 Department of Biostatistics, University of Alabama at Birmingham, Birmingham, AL 35294 USA; 6 School of Radiation Medicine and Protection and Collaborative Innovation Center of Radiation Medicine of Jiangsu Higher Education Institutions, Soochow University, Suzhou, 215006 China; 7 Department of Basic Science, Changzhou Vocational Institute of Engineering, Changzhou, Jiangsu 213164 China; 8 Department of Health management center, Hua Dong Sanatorium, Wuxi, 214065 China

**Keywords:** Retinopathy of prematurity, Risk factors

## Abstract

Elevated levels of serum uric acid (SUA) were considered to be risk factors for cardiovascular disease, it has been found to be associated with increased arteriosclerosis. The aim of this study was to explore the gender specific relationship between SUA and fundus arteriosclerosis in a healthy population. In a retrospective cross-sectional study, 23474 individuals without diabetes and hypertension were included in the present study. SUA levels were cut to four groups as Q1 to Q4, according to the quartiles. The odds ratio and 95% confidence interval of different SUA levels were estimated by a binomial logistic regression model. A restrictive cubic spline method was used to estimate the dose-response relationship between SUA and fundus arteriosclerosis. Subgroup analysis was performed to find the gender-specific association between SUA and incident fundus arteriosclerosis. In males, after adjusting for confounding factors, the highest SUA level was significantly associated with the risk of incident fundus arteriosclerosis. The OR with 95%CI for Q4 was 1.44(1.18, 1.76), Q1 as a reference. Specially, for females, SUA level was not associated with the incidence of fundus arteriosclerosis. In conclusion, elevated levels of SUA were associated with the incidence of fundus arteriosclerosis in males, but not in females.

## Introduction

Arteriosclerosis is one of the earliest signs of adverse structural and functional changes within the vessel wall^1^. Fundus arteriosclerosis is essentially retinopathy and belongs to small arteriosclerosis. Fundus arteriosclerosis has been shown to be a predictor of cardiovascular disease, coronary artery disease, new-onset hypertension, renal and cerebrovascular diseases in various populations^2–6^. In addition, fundus arteriosclerosis was the only blood vessel lesion that can be observed *in vivo* through non-invasive surgery^7^. Studies have suggested that the degree of fundus arteriosclerosis can better predict the extent of coronary artery disease^3^. Moreover, one study has shown that inflammatory response and endothelial dysfunction were associated with the pathogenesis of retinopathy^8^.

Serum uric acid (SUA) is the end product of purine metabolism, most of which is excreted in the urine by the kidneys^9^. Hyperuricemia (HUA) is a well-known cause of gout and is linked with metabolic syndrome^10^, owing to increased production of SUA coupled with excretion dysfunction. Epidemiological studies have shown that elevated levels of SUA were independent risk factors for cardiovascular events, kidney disease, metabolic syndrome, coronary heart disease, stroke and myocardial infarction^11–17^. A few experimental studies have suggested that uric acid may be involved in the formation of arteriosclerosis through multiple pathways. It may be related to arteriosclerosis by regulating important pro-inflammatory pathways^18^, may be induced by nitric oxide-related endothelial cell proliferation^19^, and may be stimulated by vascular smooth muscle proliferation^20^. Although several studies have explored the relationship between uric acid and arteriosclerosis, the results were not consistent^21–27^. Recent studies have shown that elevated SUA levels may be associated with a higher risk of increased arteriosclerosis in normotensive subjects^22^, and SUA could be used as a predictor of atherosclerosis^27^. In contrast, in a Korea study, SUA levels were not related to arteriosclerosis^21^. Animal experiment has shown that the administration of allopurinol and benzbromarone prevented the development of atherosclerosis in apoE (−/−) mice fed a uric acid-enriched diet^25^. Given these discordant results, the association between elevated uric acid and arteriosclerosis remains controversial. In addition, this association has been relatively underexplored in healthy population.

Therefore, we analyzed the relationship between SUA and funds arteriosclerosis in healthy examination population by a retrospective cross-sectional study, and explored whether there was a dose response relationship between them.

## Results

### Characteristics of study population

Baseline characteristics of participants by quartiles of SUA levels in total populations are shown in Table 1. Table 1 shows that the incidence of fundus arteriosclerosis was 9.3%. We could find that the incidence of fundus arteriosclerosis gradually increases with the increase of SUA levels (*p* for trend <0.0001). With the increase of SUA level, body mass index (BMI) gradually increased, and the number of smokers and drinkers also gradually increased. In addition, SUA levels were found significantly positive correlation with creatinine, eGFR, TG, TC and LDL. However, higher SUA levels were found to be negatively related to HDL.

**Table 1:** Baseline characteristics of participants across quartile of SUA levels in total populations.

Characteristics	Total	Q1	Q2	Q3	Q4	*p* for trend
N (participants)	23474	5866	5866	5866	5876	—
Males, n (%)	12625(53.8)	488(8.3)	2125(36.5)	4450(75.9)	5562(94.7)	<0.0001
Age, years	46(37, 54)	43(36, 51)	47(37, 54)	47(38, 55)	46(37, 54)	<0.0001
BMI, kg/m^2^	23.5(21.4, 25.5)	21.6(20.0, 23.2)	22.7(21.0, 24.6)	24.1(22.4, 26.0)	25.4(23.7, 27.3)	<0.0001
Smoking status, n (%)						<0.0001
No	17349(73.9)	5615(95.7)	4847(82.6)	3752(64.0)	3135(53.4)	
Yes	6125(26.1)	251(4.3)	1019(17.4)	2114(36.0)	2741(46.6)	
Drinking status, n (%)						<0.0001
No	13341(56.8)	5077(86.6)	4032(68.7)	2540(43.3)	1692(28.8)	
Yes	10133(43.2)	789(13.4)	1834(31.3)	3326(56.7)	4184(71.2)	
Creatinine, μmol/L	72.8(61.3, 84.4)	60.0(54.3, 66.1)	67.3(59.4, 77.9)	78.9(69.6, 87.4)	84.6(77.1, 92.2)	<0.0001
eGFR, ml/min/1.73 m^2^	96.8(83.9, 111.8)	94.7(82.8, 108.3)	94.4(81.7, 108.5)	97.2(84.4, 112.6)	101.6(87.5, 118.2)	<0.0001
Uric acid, μmol/L	339.2(274.6, 410.7)	238.3(213.8, 257.3)	307.0(290.8, 322.7)	373.0(356.0, 391.5)	460.1(432.0, 501.3)	—
TG, mmol/L	1.2(0.8, 1.9)	0.9(0.7, 1.2)	1.1(0.8, 1.5)	1.4(1.0, 2.1)	1.9(1.3, 2.8)	<0.0001
TC, mmol/L	5.0(4.4, 5.6)	4.9(4.3, 5.5)	5.0(4.4, 5.6)	5.0(4.5, 5.6)	5.1(4.6, 5.7)	<0.0001
HDL, mmol/L	1.4(1.2, 1.7)	1.7(1.4, 2.0)	1.5(1.3, 1.8)	1.3(1.1, 1.6)	1.2(1.0, 1.4)	<0.0001
LDL, mmol/L	3.0(2.5, 3.5)	2.8(2.3, 3.3)	3.0(2.5, 3.5)	3.1(2.0, 3.6)	3.2(2.7, 3.7)	<0.0001
Fundus arteriosclerosis, n (%)						
Yes	2179(9.3)	260(4.4)	486(8.3)	617(10.5)	816(13.9)	<0.0001
NO	21295(90.7)	5606(95.6)	5380(91.7)	5249(89.5)	5060(86.1)	

Notes: Serum uric acid quartiles(Q1–Q4) in total populations (Q1: ≤274.60, Q2: 274.61–339.20, Q3: 339.21–410.70, Q4: >410.70 μmol/L or Q1: ≤4.61, Q2: 4.62–5.70, Q3: 5.71–6.90, Q4: >6.90 mg/dL). BMI: body mass index; eGFR: estimated glomerular filtration rate; TG: triglycerides; TC: total cholesterol; LDL: low density lipoprotein; HDL: high density lipoprotein.

### Relationship between SUA and the incidence of fundus arteriosclerosis

As what is shown in Table 2, we found that in males the Q4 of SUA levels were independent risk of increasing incident fundus arteriosclerosis after adjustment for age, BMI, smoking, drinking, eGFR, TG, TC, LDL and HDL compared with the first quartile of SUA level. The OR with 95%CI for Q2-Q4 were 0.96(0.79, 1.16) with *p* = 0.66, 1.09(0.90, 1.32) with *p* = 0.40, and 1.44(1.18, 1.76) with *p* = 0.0003, respectively. Trend test was also significant with *p* = 0.0002. In females, the OR with 95%CI for Q2-Q4 were 0.96(0.69, 1.33) with *p* = 0.79, 1.03(0.75, 1.42) with *p* = 0.84, and 1.00(0.73, 1.38) with *p* = 0.98, respectively. Trend test was not significant with *p* = 0.85. The association between SUA and incident fundus arteriosclerosis was different in males and females. In total populations, the OR with 95%CI for Q2-Q4 were 1.08(0.89, 1.31) with *p* = 0.47, 1.03(0.84, 1.26) with *p* = 0.79, 1.40(1.13, 1.75) with *p* = 0.0027. *p* for trend = 0.0010 (Supplementary Table S1).

**Table 2:** OR and 95% CI for changes in SUA for fundus arteriosclerosis incidence according to quartiles of SUA in males and females.

Variable	Model 1^a^		Model 2^b^		Model 3^c^	
	OR, 95%CI	*p* value	OR, 95%CI	*p* value	OR, 95%CI	*p* value
Males						
Q1	1.00(refer)	—	1.00(refer)	—	1.00(refer)	—
Q2	0.95(0.81, 1.11)	0.48	0.95(0.79, 1.15)	0.61	0.96(0.79, 1.16)	0.66
Q3	1.05(0.90, 1.22)	0.55	1.09(0.90, 1.32)	0.37	1.09(0.90, 1.32)	0.40
Q4	1.32(1.14, 1.53)	0.0002	1.47(1.21, 1.77)	<0.0001	1.44(1.18, 1.76)	0.0003
*p* for trend	<0.0001		<0.0001		0.0002	
Females						
Q1	1.00(refer)	—	1.00(refer)	—	1.00(refer)	—
Q2	1.33(1.00, 1.77)	0.05	0.97(0.70, 1.35)	0.85	0.96(0.69, 1.33)	0.79
Q3	1.94(1.49, 2.53)	<0.0001	1.05(0.77, 1.44)	0.77	1.03(0.75, 1.42)	0.84
Q4	3.14(2.45, 4.02)	<0.0001	1.06(0.78, 1.43)	0.73	1.00(0.73, 1.38)	0.98
*p* for trend	<0.0001		0.59		0.85	

Notes: Serum uric acid quartiles(Q1–Q4) in males (Q1: ≤349.30, Q2: 349.31–398.90, Q3: 398.91–453.40, Q4: >453.40 μmol/L or Q1: ≤5.87, Q2: 5.88–6.70, Q3: 6.71–7.62, Q4: >7.62 mg/dL); in females (Q1:≤237.30, Q2: 237.31–275.30, Q3: 275.31–316.60, Q4: >316.60 μmol/L or Q1: ≤3.99, Q2: 4.00–4.63, Q3: 4.64–5.32, Q4: >5.32 mg/dL). BMI: body mass index; eGFR: estimated glomerular filtration rate; TG: triglycerides; TC: total cholesterol; LDL: low density lipoprotein; HDL: high density lipoprotein.

^a^Model 1: unadjusted. ^b^Model 2: adjusted for age, BMI, smoking and drinking.

^c^Model 3: adjusted for age, BMI, smoking, drinking, eGFR, TG, TC, LDL and HDL.

**Figure 1:**
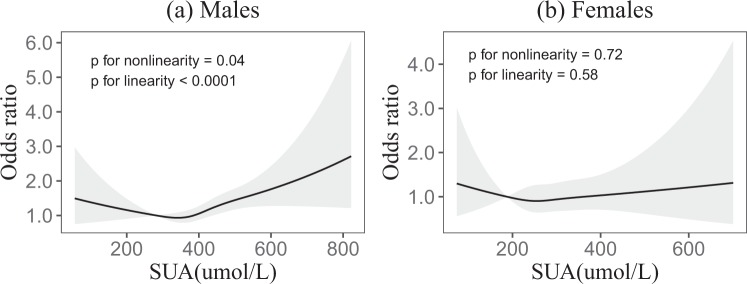
Association of SUA with the incidence of fundus arteriosclerosis according to restricted cubic spline regressions using four knots in males and females (percentiles 5, 35, 65 and 95), with the reference point set at percentile 12.5. (**a,b**) Odds ratios were adjusted for age, BMI, smoking, drinking, eGFR, TG, TC, LDL and HDL, respectively.

Figure 1 shows the dose–response relationships between SUA and the risk of incident fundus arteriosclerosis. The association between SUA and the incidence of fundus arteriosclerosis was modeled through multivariable-adjusted spline regression models with four knots, and revealed linear dose-response relationships between uric acid and incident fundus arteriosclerosis in males (*p* < 0.0001). But there was no significant linear dose-response relationship in females (*p* = 0.58). There were liner dose-response relationships between SUA and incident fundus arteriosclerosis in total (*p* < 0.0001) (see Supplementary Fig. S1).

### The BMI subgroup analysis for association of SUA with incident fundus arteriosclerosis

We subsequently performed subgroups analysis by major characteristics of the study population. Table 3 shows the analysis of relationship between SUA and incident fundus arteriosclerosis by BMI subgroup analysis in different groups. We could find that after adjustment for age, smoking, drinking, eGFR, TG, TC, LDL and HDL, in male subgroup with BMI ≤ 25 kg/m^2^, the OR for incident fundus arteriosclerosis from the second to the highest SUA quartile were 1.15(0.88, 1.50) with *p* = 0.31, 1.23(0.93, 1.63) with *p* = 0.15, 1.96(1.45, 2.64) with *p* < 0.0001, respectively. *p* for trend < 0.0001. In male subgroup with BMI > 25 kg/m^2^, the OR for incident fundus arteriosclerosis from the second to the highest SUA quartile were 0.88(0.67, 1.17) with *p* = 0.39, 1.13(0.86, 1.48) with *p* = 0.38, 1.52(1.16, 1.98) with *p* = 0.0020, respectively. *p* for trend <0.0001. In female subgroup with BMI ≤ 25 kg/m^2^, the OR for incident fundus arteriosclerosis from the second to the highest SUA quartile were 0.92(0.64, 1.33) with *p* = 0.66, 0.98(0.68, 1.40) with *p* = 0.90, 1.23(0.86, 1.76) with *p* = 0.25, respectively. *p* for trend = 0.17. In female subgroup with BMI > 25 kg/m^2^, the OR for incident fundus arteriosclerosis were from the second to the highest SUA quartile were 1.64(0.77, 3.50) with *p* = 0.20, 1.80(0.90, 3.61) with *p* = 0.10, 1.32(0.67, 2.59) with *p* = 0.42, respectively. *p* for trend = 0.94. As can be seen from the Table 3, in the BMI subgroup analysis, there was a gender difference in the relationship between uric acid and incident fundus arteriosclerosis, which was statistically significant in males and not statistically significant in females. In total subgroup with BMI ≤ 25 kg/m^2^, the OR for incident fundus arteriosclerosis from the second to the highest SUA quartile were 1.04(0.83, 1.32) with *p* = 0.72, 1.26(0.98, 1.63) with *p* = 0.08, 1.76(1.32, 2.35) with *p* = 0.0001, respectively. *p* for trend < 0.0001. In total subgroup with BMI > 25 kg/m^2^, the OR for incident fundus arteriosclerosis from the second to the highest SUA quartile were 1.32(0.92, 1.90) with *p* = 0.14, 1.05(0.73, 1.50) with *p* = 0.81, 1.67(1.15, 2.41) with *p* = 0.01, respectively. *p* for trend = 0.0010 (Supplementary Table S2).

**Table 3:** In the BMI subgroups, univariate and multivariate logistic regression analysis of the relationship between uric acid and fundus arteriosclerosis, in males and females.

Variable		Model 1^a^		Model 2^b^		Model 3^c^	
		OR, 95%CI	*p* value	OR, 95%CI	*p* value	OR, 95%CI	*p* value
males							
BMI							
≤25	Q1	1.00(refer)		1.00(refer)	—	1.00(refer)	—
	Q2	0.96(0.78, 1.19)	0.72	1.17(0.90, 1.52)	0.25	1.15(0.88, 1.50)	0.31
	Q3	1.01(0.80, 1.26)	0.97	1.27(0.96, 1.66)	0.09	1.23(0.93, 1.63)	0.15
	Q4	1.32(1.05, 1.66)	0.02	2.04(1.54, 2.71)	<0.0001	1.96(1.45, 2.64)	<0.0001
*p* for trend		0.03		<0.0001		<0.0001	
>25	Q1	1.00(refer)	—	1.00(refer)	—	1.00(refer)	—
	Q2	0.79(0.63, 1.00)	0.05	0.86(0.65, 1.13)	0.29	0.88(0.67, 1.17)	0.39
	Q3	0.83(0.67, 1.04)	0.10	1.11(0.85, 1.44)	0.45	1.13(0.86, 1.48)	0.38
	Q4	0.93(0.76, 1.15)	0.50	1.49(1.16, 1.91)	0.0020	1.52(1.16, 1.98)	0.0020
*p* for trend		0.96		<0.0001		<0.0001	
females							
BMI							
≤25	Q1	1.00(refer)		1.00(refer)	—	1.00(refer)	—
	Q2	1.20(0.87, 1.66	0.27	0.97(0.67, 1.39)	0.85	0.92(0.64, 1.33)	0.66
	Q3	1.56(1.14, 2.13)	0.01	1.06(0.74, 1.52)	0.75	0.98(0.68, 1.40)	0.90
	Q4	2.73(2.04, 3.66)	<0.0001	1.44(1.03, 2.02)	0.04	1.23(0.86, 1.76)	0.25
*p* for trend		<0.0001		0.0178		0.1741	
>25	Q1	1.00(refer)	—	1.00(refer)	—	1.00(refer)	—
	Q2	1.56(0.85, 2.86)	0.15	1.75(0.84, 3.68)	0.14	1.64(0.77, 3.50)	0.20
	Q3	1.83(1.05, 3.17)	0.03	1.78(0.90, 3.50)	0.10	1.80(0.90, 3.61)	0.10
	Q4	1.67(0.99, 2.82)	0.06	1.40(0.74, 2.66)	0.31	1.32(0.67, 2.59)	0.42
*p* for trend		0.12		0.84		0.94	

Notes: Serum uric acid quartiles(Q1–Q4) in males (Q1: ≤349.30, Q2: 349.31–398.90, Q3: 398.91–453.40, Q4: >453.40 μmol/L or Q1: ≤5.87, Q2: 5.88–6.70, Q3: 6.71–7.62, Q4: >7.62 mg/dL); in females (Q1: ≤237.30, Q2: 237.31–275.30, Q3: 275.31–316.60, Q4: >316.60 μmol/L or Q1: ≤3.99, Q2: 4.00–4.63, Q3: 4.64–5.32, Q4: >5.32 mg/dL). BMI: body mass index; eGFR: estimated glomerular filtration rate; TG: triglycerides; TC: total cholesterol; LDL: low density lipoprotein; HDL: high density lipoprotein.

^a^Model 1: unadjusted. ^b^ Model 2: adjusted for age, smoking and drinking.

^c^Model 3: adjusted for age, smoking, drinking, eGFR, TG, TC, LDL and HDL.

**Figure 2:**
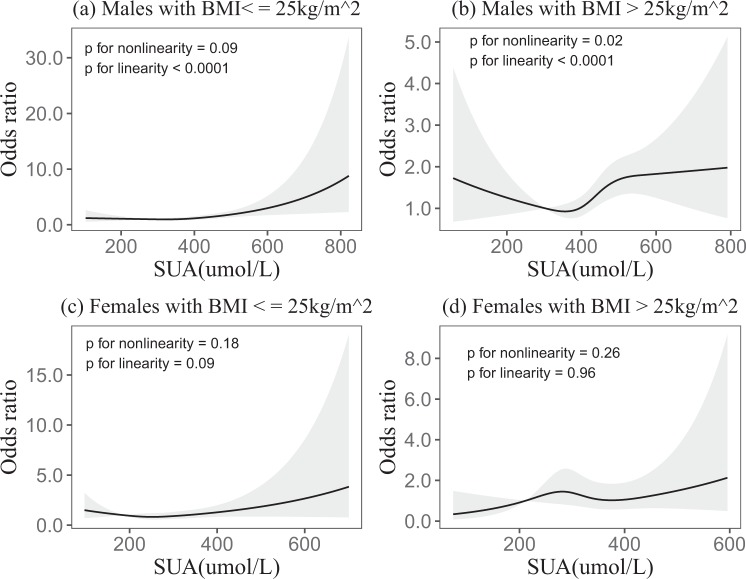
In the BMI subgroups, association of SUA with the incidence of fundus arteriosclerosis according to restricted cubic spline regressions using four knots in males and females (percentiles 5, 35, 65 and 95), with the reference point set at percentile 12.5. (**a–d**) Odds ratios were adjusted for age, smoking, drinking, eGFR, TG, TC, LDL and HDL, respectively.

In the BMI subgroup analysis, which revealed liner dose-response relationships between SUA and incident fundus arteriosclerosis in males BMI ≤ 25 kg/m^2^ and BMI > 25 kg/m^2^ (*p* < 0.0001 and *p* < 0.0001). But there were no linear dose-response relationships in females BMI ≤ 25 kg/m^2^ and BMI > 25 kg/m^2^ (*p* = 0.09 and *p* = 0.96) (Fig. 2). The BMI subgroup analysis, which revealed liner dose-response relationships between SUA and incident fundus arteriosclerosis in total BMI ≤ 25 kg/m^2^ and BMI > 25 kg/m^2^ (*p* < 0.0001 and *p* < 0.0001) (see supplementary Fig. S2).

### The age Subgroup analysis for association of SUA with incident fundus arteriosclerosis

Table 4 shows that relationship between uric acid and incident fundus arteriosclerosis by age subgroup in different groups. We could observe that in male subgroup with age ≤50 years, the OR for incident fundus arteriosclerosis from the second to the highest SUA quartile were 1.09(0.63, 1.89) with *p* = 0.75, 0.99(0.57, 1.71) with *p* = 0.97, 1.41(0.85, 2.36) with *p* = 0.19, respectively. *p* for trend = 0.15. In male subgroup with age >50 years, the OR for incident fundus arteriosclerosis from the second to the highest SUA quartile were 0.99(0.82, 1.18) with *p* = 0.89, 1.15(0.96, 1.37) with *p* = 0.14, 1.56(1.29, 1.86) with *p* < 0.0001, respectively. *p* for trend < 0.0001. In female subgroup with age ≤50 years, the OR for incident fundus arteriosclerosis from the second to the highest SUA quartile were 0.60(0.24, 1.48) with *p* = 0.26, 0.83(0.37, 1.85) with *p* = 0.64, 0.53(0.23, 1.24) with *p* = 0.14, respectively. *p* for trend = 0.25. In female subgroup with age >50 years, the OR for incident fundus arteriosclerosis from the second to the highest SUA quartile were 0.92(0.66, 1.28) with *p* = 0.61, 0.92(0.67, 1.26) with *p* = 0.61, 0.87(0.64, 1.19) with *p* = 0.39, respectively. *p* for trend = 0.43. As can be seen from the Table 4, in the age subgroup analysis, there was a gender difference in the relationship between uric acid and incident fundus arteriosclerosis, which was statistically significant in males age >50 years and not statistically significant in females. In total age ≤50 years, the OR for incident fundus arteriosclerosis from the second to the highest SUA quartile were 1.08(0.62, 1.87) with *p* = 0.78, 0.99(0.55, 1.78) with *p* = 0.97, 1.23(0.67, 2.25) with *p* = 0.51, respectively. *p* for trend = 0.41. In total age >50 years, the OR for incident fundus arteriosclerosis from the second to the highest SUA quartile were 1.23(1.03, 1.47) with *p* = 0.02, 1.29(1.07, 1.56) with *p* = 0.01, 1.80(1.47, 2.21) with *p* < 0.0001, respectively. *p* for trend < 0.0001 (Supplementary Table S3).

**Table 4:** In the age subgroups, univariate and multivariate logistic regression analysis of the relationship between uric acid and fundus arteriosclerosis, in males and females.

		Model 1^a^		Model 2^b^		Model 3^c^	
		OR, 95%CI	*p* value	OR, 95%CI	*p* value	OR, 95%CI	*p* value
males							
Age							
≤50	Q1	1.00(refer)		1.00(refer)	—	1.00(refer)	—
	Q2	1.42(0.83, 2.45)	0.20	1.18(0.68, 2.03)	0.56	1.09(0.63, 1.89)	0.75
	Q3	1.58(0.93, 2.68)	0.09	1.11(0.65, 1.90)	0.71	0.99(0.57, 1.71)	0.97
	Q4	3.11(1.93, 5.03)	<0.0001	1.73(1.05, 2.85)	0.03	1.41(0.85, 2.36)	0.19
*p* for trend		<0.0001		0.02		0.15	
>50	Q1	1.00(refer)	—	1.00(refer)	—	1.00(refer)	—
	Q2	1.00(0.84, 1.20)	0.99	1.00(0.84, 1.20)	0.98	0.99(0.82, 1.18)	0.89
	Q3	1.16(0.98, 1.39)	0.09	1.17(0.98, 1.40)	0.08	1.15(0.96, 1.37)	0.14
	Q4	1.59(1.33, 1.89)	<0.0001	1.60(1.34, 1.91)	<0.0001	1.56(1.29, 1.86)	<0.0001
*p* for trend		<0.0001		0.03		<0.0001	
females							
Age							
≤50	Q1	1.00(refer)	—	1.00(refer)	—	1.00(refer)	—
	Q2	0.87(0.36, 2.11)	0.76	0.76(0.31, 1.84)	0.53	0.60(0.24, 1.48)	0.26
	Q3	1.69(0.78, 3.64)	0.18	1.12(0.51, 2.45)	0.78	0.83(0.37, 1.85)	0.64
	Q4	2.25(1.07, 4.73)	0.03	0.79(0.35, 1.81)	0.58	0.53(0.23, 1.24)	0.14
*p* for trend		0.01		0.78		0.25	
>50	Q1	1.00(refer)	—	1.00(refer)	—	1.00(refer)	—
	Q2	1.15(0.84, 1.58)	0.39	1.01(0.73, 1.40)	0.95	0.92(0.66, 1.28)	0.61
	Q3	1.36(1.01, 1.83)	0.04	1.10(0.81, 1.49)	0.55	0.92(0.67, 1.26)	0.61
	Q4	1.89(1.43, 2.50)	<0.0001	1.25(0.93, 1.67)	0.14	0.87(0.64, 1.19)	0.39
*p* for trend		<0.0001		0.07		0.43	

Notes: Serum uric acid quartiles(Q1–Q4) in males (Q1: ≤349.30, Q2: 349.31–398.90, Q3: 398.91–453.40, Q4: >453.40 μmol/L or Q1: ≤5.87, Q2: 5.88–6.70, Q3: 6.71–7.62, Q4: >7.62 mg/dL); in females (Q1: ≤237.30, Q2: 237.31–275.30, Q3: 275.31–316.60, Q4: >316.60 μmol/L or Q1: ≤3.99, Q2: 4.00–4.63, Q3: 4.64–5.32, Q4: >5.32 mg/dL). BMI: body mass index; eGFR: estimated glomerular filtration rate; TG: triglycerides; TC: total cholesterol; LDL: low density lipoprotein; HDL: high density lipoprotein.

^a^Model 1: unadjusted. ^b^Model 2: adjusted for BMI, smoking and drinking.

^c^Model 3: adjusted for BMI, smoking, drinking, eGFR, TG, TC, LDL and HDL.

**Figure 3:**
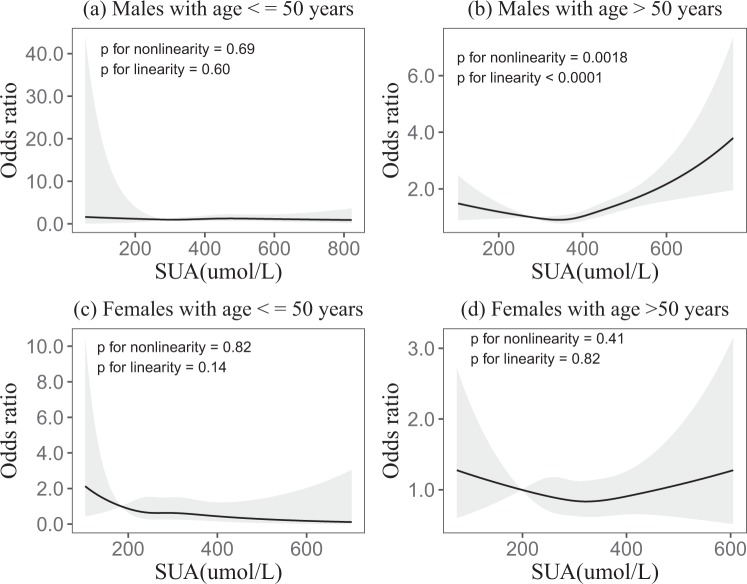
In the age subgroups, association of SUA with the incidence of fundus arteriosclerosis according to restricted cubic spline regressions using four knots in males and females (percentiles 5, 35, 65 and 95), with the reference point set at percentile 12.5. (**a–d**) Odds ratios were adjusted for BMI, smoking, drinking, eGFR, TG, TC, LDL and HDL, respectively.

In the age subgroup analysis, which revealed linear dose-response relationships between uric acid and fundus arteriosclerosis in males age >50 years (*p* < 0.0001). But there were no linear dose-response relationships in males age ≤50 years and females age ≤50 years and females age >50 years (*p* = 0.60, *p* = 0.14 and *p* = 0.82) (Fig. 3). In the age subgroup analysis, which revealed liner dose-response relationships between uric acid and fundus arteriosclerosis in total age >50 years (p < 0.0001), but there was no linear dose-response relationship in total age ≤50 years (p = 0.89) (see Supplementary Fig. S3).

## Discussion

The results of this study showed that 9.3% of people without diabetes and hypertension showed signs of fundus arteriosclerosis. In addition, the incidence of fundus arteriosclerosis in males and females were 12.3% and 5.8%, respectively. The incidence of fundus arteriosclerosis was similar to many previous studies and should be of great concern^28–31^. In this study, we observed a particularly important result that there was a gender-specific association between serum uric acid and fundus arteriosclerosis, and there was a significant dose-response relationship between higher SUA levels and the incidence of fundus arteriosclerosis.

Fundus arteriosclerosis is a microvascular disease that belongs to arteriosclerosis. To rule out the effects of traditional factors on arteriosclerosis, this study excluded traditional cardiovascular risk factors such as diabetes and hypertensions, and screened for a healthy physical examination. Our study showed that the association between SUA and fundus arteriosclerosis was statistically significant in males without diabetes or hypertension. Chen’s^32^ study showed that uric acid was positively associated with aortic stiffness in men. Previous studies have shown that uric acid was found to be an independent risk factor for carotid atherosclerosis in men without metabolic syndrome^33^. When the SUA level was above 5.2 mg/dl, arterial stiffness was increased in healthy Korean men^34^. Some longitudinal studies have shown that uric acid was a risk factor for arteriosclerosis, the relationship was more pronounced in men^35^. Shin’s^21^ and Tomiyama’s^36^ studies showed an insignificant relationship between uric acid and arterial stiffness in women with normal uric acid level. Recently, a cohort study disclosed a significant independent relationship between increased SUA and increased pulse wave velocity (PWV) over time, this association was restricted to men, and possibly explained by the higher SUA levels in men than in women throughout their lifespan^37^. Ishizaka, N., *et al*. confirmed that in both genders, SUA level was associated with arterial stiffness, and was in part independent of other conventional risk factors for atherosclerosis and metabolic syndrome^38^. However, Yuan, Y., *et al*. showed that in the Chinese coastal female populations, the association of high SUA and increased arterial stiffness was dependent on the coexistence of at least one cardiovascular risk factor, especially dyslipidemia^39^.

Some studies also showed that there was a gender-specific association between higher uric acid and coronary artery disease, only statistically different among women^40^. It should be noted that our results were inconsistent with those of some predecessors, and we concluded that elevated uric acid was associated with fundus arteriosclerosis in males, but not in females. Possible explanations were as follows. First, different subjects were selected, and dietary habits and genes of different populations could also cause differences in the incidence of fundus arteriosclerosis. Secondly, uric acid levels were generally higher in males than in females^37^. However, this gender difference has often been attributed to the role of estrogen in females to promote uric acid excretion before menopause^41^ and possibly to impair renal clearance and excess uric acid secretion in male patients, especially in the presence of visceral fat obesity or metabolic syndrome^42^, the pathophysiology and clinical significance of this finding have not been clearly elucidated^40^. In addition, the incidence of fundus arteriosclerosis in males was higher than that of females. The mechanisms of gender difference in the effect of uric acid on fundus arteriosclerosis are still unclear, and require further research.

In the subgroup analysis of age >50 years, SUA levels related to Q4 quartile were associated with the incidence of fundus arteriosclerosis in males, but not in females. Recently, studies have shown that with the increase of age, male individuals with higher SUA values had an accelerated increase in PWV, which was not related to major confounding factors such as age, blood pressure, renal function, and metabolic measurement^37^. A prospective cohort study of adults in northern China aged 40 years and older showed that uric acid was associated with arteriosclerosis, but not in women^35^. Previous studies have shown that SUA was an independent risk factor for arteriosclerosis in women older than 55 years^1^, but our studies showed that there were not statistically significant in females. The results of this study are inconsistent with previous results. The mechanism of SUA affecting fundus arteriosclerosis is complex and unclear, so this relationship needs further study.

A recent cross-sectional analysis of adults aged from 30 to 45 showed that the relationship between SUA and early atherosclerosis has been eliminated after BMI adjustment^43^. A longitudinal study has shown that SUA might be an early biomarker for subclinical atherosclerosis in young adults; starting in early middle age, SUA predicted subclinical atherosclerosis independently of BMI^44^. This study showed that in males, regardless of the extent of BMI, the highest SUA was associated with the incidence of fundus arteriosclerosis, independent of the effects of BMI, and consistent with previous studies.

Fundus Arteriosclerosis was the result of a complex interaction between the structural and geometric properties of the arterial wall and the dynamic effects of inflation pressure^37^. There are several aspects can explain the mechanism of uric acid and fundus arteriosclerosis. Uric acid may stimulate the inflammatory pathways^18^ and promote vascular smooth muscle cells (VSMC) proliferation and oxidative stress by vascular renin-angiotensin system^45^. In human vascular cells and endothelial cells, SUA has been also found to upregulate c-reactive protein and was directly involved in pro-inflammatory and atherogenic processes^46,47^, then leads to endothelial cell damage. Uric acid also promotes endothelial dysfunction through inactivation of NO and impairment of endothelial cells proliferation^46^. Moreover, urate crystals are deposited on the arterial wall, which causes inflammation, stimulates mast cells, induces lipid infiltration, and further destroys the intimal cells of the arteries, thereby blocking the metabolism of apolipoprotein B (apoB) and eventually leading to the aggravation of atherosclerosis^27^. Uric acid has a dual role, in addition to an oxidative effect, it also has an antioxidant effect^48^. Although uric acid has a protective effect, elevated uric acid levels are often associated with increased risk of cardiovascular disease and death^12^. Our study also showed that the association between elevated uric acid and fundus arteriosclerosis was statistically significant. There are two possible reasons that can explain our findings: either uric acid is causing arteriolosclerosis or it is generated to protect against arteriolosclerosis. Our research suggested that there was a relationship between elevated uric acid and fundus arteriosclerosis. However, the relationship between uric acid and fundus arteriosclerosis is unclear, and further research is needed.

It is worth noting that the dose-response relationship between SUA levels and the incidence of fundus arteriosclerosis is seen in males. Although the underlying pathophysiological mechanisms are unclear, excluding interference from traditional risk factors leaving significant deleterious effects in relatively healthy adults. The role of uric acid in atherosclerosis might be related to other cardiovascular risk factors, such as hypertension and metabolic syndrome^23^. However, some studies have shown that SUA was associated with increased aortic stiffness in Chinese adults and was not associated with conventional cardiovascular risk factors^24^. This finding was consistent with previous studies, which were associated with increased levels of SUA and were not associated with traditional cardiovascular risk factors^24^. The results of this study suggested that elevated levels of SUA were associated with the incidence of fundus arteriosclerosis in subjects without diabetes and hypertension, especially in males. Therefore, more attention should be paid to this type of population in health screening. Preventing the incident of fundus arteriosclerosis in advance, on the one hand, preventing visual impairment, on the other hand, it can provide timely and effective basis for clinicians to further assist in the diagnosis of cardiovascular and cerebrovascular diseases.

In this study, we used uric acid quartiles for statistical analysis. At the same time, we included uric acid as a continuous variable in the logistic model analysis, and the results were consistent with those in the article. We found that in males SUA levels were independent risk of increasing incident fundus arteriosclerosis after adjusting for confounding factors, the OR with 95%CI for SUA was 1.002(1.001, 1.003) with *p* < 0.0001. In females, the OR with 95%CI for SUA was 1.000(0.999, 1.002) with *p* = 0.59. We could also find that there was a gender specific association between uric acid and fundus arteriosclerosis, which was statistically significant in males, but not in females. (see supplementary Table S4). In the analysis of uric acid as a continuous variable, the OR value of the relationship between uric acid and fundus arteriosclerosis was generally small. In order to research the association between uric acid and fundus arteriosclerosis more deeply, we have used uric acid quartile as the study variable to analyze, and also to be more clearly describe the effect of uric acid increase on fundus arteriosclerosis.

In addition, we used the clinical reference value of uric acid as an independent variable for statistical analysis, and we found that the number of males and females were relatively small (54 and 38 respectively) at the low levels of uric acid. (see supplementary Table S5). The statistical power will be reduced if the number of people was small. Then, we combined the number of people with low and normal levels of uric acid, and uric acid was divided into two categories for statistical analysis (Q1: less than high level; Q2: more than high level) (See Supplementary Table S6). After adjusted for other confounding factors, elevated levels of SUA were associated with the incidence of fundus arteriosclerosis in males, but not in females. This result was consistent with our previous findings. If we take the clinical reference value of uric acid as the study variable, more sample size is needed to confirm the relationship between uric acid and fundus arteriosclerosis.

Moreover, we divided the independent variables into those with or without hyperuricemia by using clinical reference values of hyperuricemia, and conducted a logistics regression analysis. We found that the association between hyperuricemia and fundus arteriosclerosis was statistically significant in males, but not in females. (See Supplementary Table S7). This result was consistent with our previous results. There was a gender difference in the relationship between elevated uric acid and fundus arteriosclerosis, the mechanism of which was not clear, and more research was needed to confirm this.

There were still some limitations in this research. Firstly, this was a retrospective cross-sectional study that did not demonstrate the causal relationship between SUA and fundus arteriosclerosis. Secondly, this study was a retrospective study, some potential factors could not be controlled. Nevertheless, the advantage of our research was that the sample size was large, excluded those with hypertension, diabetes and the total populations, males and females were analyzed separately, which ensured sufficient parameters and accurate results, and drawn a solid conclusion that there was a difference between males and females. Finally, a future prospective study is needed to determine whether the SUA level is an independent predictor of long-term fundus arteriosclerosis outcomes, and whether its reduction could reduce the occurrence of adverse fundus arteriosclerosis events.

## Materials and methods

### Study population

**Figure 4:**
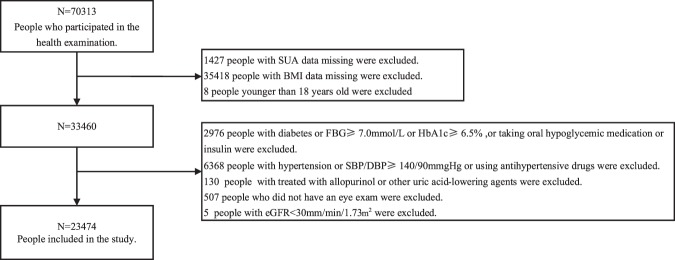
Describe the sample flow chart for screening studies for statistical analysis.

This study analyzed data from Hua Dong Sanatorium. A total of 70,313 non-manual workers underwent health examination in 2018. To avoid confounding factors, subjects with a history of hypertension, diabetes, and those taking medications influencing blood pressure, plasma glucose, lipid profile and uric acid were all excluded. In addition, subjects with blood pressure 140/90 mmHg, fasting plasma glucose≥7.0 mmol/L, haemoglobinA1c (HbA1c) ≥6.5%, and estimated glomerular filtration rate (eGFR) <30 ml/min/1.73 m^2^ were also excluded. A total of 23474 (12625 males and 10849 females) aged 18–91 years were included in this study. Data clean steps were presented in Fig. 4. This study was approved by the Ethical Committee and the Institutional Review Board of Hua Dong Sanatorium, Wuxi. The informed consent was waived and the need for waiving the informed consent was also supported by the Ethical Committee. All methods were performed in accordance with the Declaration of Helsinki and the relevant guidelines.

### Assessment of SUA

SUA levels were determined at the Hua Dong Sanatorium laboratory with Enzymatic methods by AU 5400 BECKMAN COULTER. Total population SUA quartile (Q1–Q4) were ≤274.60, 274.61–339.20, 339.21–410.70, >410.70 μmol/L, respectively. SUA levels were categorized into four groups(Q1–Q4) according to the quartiles of gender-specific distribution: ≤349.30, 349.31–398.90, 398.91–453.40, >453.40 μmol/L for males; ≤237.30, 237.31–275.30, 275.31–316.60, >316.60 μmol/L for females.

### Questionnaire data

A standard questionnaire was conducted by trained staff to obtain information about demographic characteristics (age, gender) and lifestyle risk factors such as smoking, drinking. The interview included questions related to the diagnosis and treatment of diabetes, hypertension, cardiovascular events. Smoking habit was categorized as current smoking or nonsmoking (including never smoke or ex-smokers). Drinking was categorized as current drinking or nondrinking (including never drink or ex-drinkers).

### Anthropometrics measurement

When measuring height and weight, the population stood upright, wearing a single layer of clothing, without wearing a hat or shoes. Body mass index (BMI) was calculated as weight in kilogram divided by the square of height in meter(kg/m^2^). Hypertension was defined as blood pressure ≥140/90 mmHg, or a self-reported physician diagnosed with hypertension, or a diagnosis code in the system, or an individual currently using antihypertensive drugs^49^. Diabetes was defined as fasting glucose level ≥7.0 mmol/L, or a self-reported physician diagnosis of diabetes, or a diagnosis code in the system, or taking oral hypoglycemic medication or insulin^50^.

### Laboratory testing

After fasting for at least 8 hours, blood samples were collected from the anterior cubital vein in the morning. Fasting blood glucose levels were determined by a colorimetry method (AU5400, BECKMAN COULTER). Enzymatic methods were used to measure creatinine, total cholesterol (TC), triglyceride (TG), low-density lipoprotein (LDL), high-density lipoprotein (HDL) (AU5400, BECKMAN COULTER), and haemoglobinA1c (HbA1c) was measured with high pressure liquid chromatography (HPLC) by Tosoh Automated Glycohemoglobin Analyzer HLC-723G8. The eGFR was estimated by using the Modification of Diet in Renal Disease (MDRD)-4 equation: eGFR = 186 × serum creatinine^−1.154^ × age^−0.203^ × 0.742(in women) × 1.212^51^.

### Diagnosis of fundus arteriosclerosis

An eye exam was performed by a trained ophthalmologist. Fundus photography was completed following a standardized Protocol^52^. Both eyes of each participant were photographed with a 45° digital nonmydriatic camera and were seated in a dark room. The analysis of fundus arteries was conducted with the non-dilated fundus camera TRC.NW400TOPCON. All images were evaluated by trained ophthalmologist.

Subjects were classified with regard to their retinal photography based on the Keith-Wagener-Barker classification^53^. Grade 1 is defined as retinal artery spasm or mild sclerosis. Grade 2 is defined as moderate to marked sclerosis of the retinal arterioles, arteriovenous intersection can present different degrees of pathological changes, or arteriosclerotic retinopathy or thrombosis of retinal veins. Grade 3 is defined as angiospastic retinopathy, characterized by edema, cotton-wool patches, and hemorrhages in the retina, in addition to marked sclerosis of the retinal arterioles. Grade 4 is defined as measurable edema of the disks in addition to grade 3 pictures^54^. In the present study, subjects without retinopathy were graded as normal, and subjects with any of the four grades is considered to be fundus arteriosclerosis.

### Statistical analyses

All participants were classified according to the quartiles of SUA. The baseline characteristics were compared across the SUA quartiles of the total populations. Baseline characteristics of the participants were reported as medians (quartile intervals) for continuous variables (the continuous variables were not normally distributed) and numbers (percentages) for categorical variables. Kruskal–Wallis tests were employed to compare continuous variables, whereas categorical variables were compared by χ^2^ trend tests. Univariate and multivariate logistic regression analysis were used to estimate odds ratios (ORs) and 95% confidence intervals (CIs) for changes in SUA in total populations, males and females.

To detect any possible linear or non-linear dependency in regression models and to allow for flexible interpretation of the relationship between continuous covariates and study outcomes, continuous changes in SUA were assessed through shape-restricted cubic spline regression models with knots at the 5^th^, 35^th^, 65^th^ and 95^th^ percentiles, and with the 12.5^th^ percentile as the reference category^55^.

To examine the consistency of the observed association between SUA and fundus arteriosclerosis, we performed subgroup analyses of participants according to BMI ( ≤ 25, >25 kg/m^2^), age (≤50, >50 years) in different populations. Statistical analyses were performed with SAS version 9.4 (SAS Institute, Cary, NC, USA). A 2-sided *p* value < 0.05 was considered to be statistically significant.

## Supplementary information

Supplementary Information.

## Data Availability

The datasets generated during and/or analysed during the current study are not publicly available due to cooperation unit needs to be confidential, but are available from the corresponding author on reasonable request.
