# Supplementary material for: Gender-Specific Association between Serum Uric Acid and Incident Fundus Arteriosclerosis in Chinese Population: A Retrospective Cross-Sectional Study
Source: Sci Rep. 2020 May 25;10:8595. doi: 10.1038/s41598-020-65575-z (PMC7248060; doi:10.1038/s41598-020-65575-z)
Supplement: Supplementary file 1 — Supplementary Information. [file 41598_2020_65575_MOESM1_ESM.pdf]

Gender-Specific Association between Serum Uric Acid and Incident Fundus Arteriosclerosis in Chinese Population: A Retrospective Cross-Sectional Study

Qianqian Liu[1,2#], Chunxing Liu[3#], Yonghui Gao[3], Xinyan Zhang[4], Nengjun Yi[5], Jianping Cao[6], Yamin Wang[7], Yongbin Jiang[8*], and Zaixiang Tang[1,2*]

1, Department of Biostatistics, School of Public Health, Medical College of Soochow University, Suzhou 215123, China.
2, Jiangsu Key Laboratory of Preventive and Translational Medicine for Geriatric Diseases, Medical College of Soochow University, Suzhou, 215123, China.
3, Department of Laboratory, Hua Dong Sanatorium, Wuxi, 214065, China.
4, Department of Biostatistics, Jiann-Ping Hsu College of Public Health, Georgia Southern University, Statesboro, GA 30458, USA.
5, Department of Biostatistics, University of Alabama at Birmingham, Birmingham, AL 35294, USA.
6, School of Radiation Medicine and Protection and Collaborative Innovation Center of Radiation Medicine of Jiangsu Higher Education Institutions, Soochow University, Suzhou 215006, China.
7, Department of Basic Science, Changzhou Vocational Institute of Engineering, Changzhou, Jiangsu 213164, China.
8, Department of Health management center, Hua Dong Sanatorium, Wuxi, 214065, China.

# Qianqian Liu and Chunxing Liu contributed equally to this work.

*** Correspondence author:**

Zaixiang Tang

Department of Biostatistics

School of Public Health

Medical College of Soochow University, Suzhou, 215123, China Phone:

0512-65883227

Fax: 0512-65883323

Email: tangzx@suda.edu.cn

Yongbin Jiang
Department of Health management
center Hua Dong Sanatorium, Wuxi,
214065, China Email:
1791343519@qq.com

Supplementary Table S1. OR and 95% CI for changes in SUA for fundus arteriosclerosis incidence according to quartiles of SUA in total populations.

Variable	Model 1[a]		Model 2[b]		Model 3[c]	
	OR, 95%CI	*p* value	OR, 95%CI	*p* value	OR, 95%CI	*p* value
Total						
Q1	1.00(refer)	-	1.00(refer)	-	1.00(refer)	-
Q2	1.95(1.68, 2.27)	<0.0001	1.08(0.89, 1.31)	0.46	1.08(0.89, 1.31)	0.46
Q3	2.49(2.15, 2.88)	<0.0001	1.04(0.85, 1.27)	0.72	1.03(0.84, 1.26)	0.79
Q4	3.44(2.99, 3.97)	<0.0001	1.45(1.17, 1.79)	0.0007	1.40(1.13, 1.75)	0.0027
p for trend	<0.0001		0.0001		0.0010	

Notes: Cut-points: Total (Q1: ≤274.60, Q2: 274.61-339.20, Q3: 339.21-410.70, Q4: >410.70 μmol/L or Q1: ≤4.61, Q2: 4.62-5.70, Q3: 5.71-6.90, Q4: >6.90mg/dL). BMI: body mass index; eGFR: estimated glomerular filtration rate; TG: triglycerides; TC: total cholesterol; LDL: low density lipoprotein; HDL: high density lipoprotein.

a Model 1: unadjusted. [b] Model 2: adjusted for age, gender, BMI, smoking and drinking.

[c] Model 3: adjusted for age, gender, BMI, smoking, drinking, eGFR, TG, TC, LDL and HDL.

Supplementary Table S2. In the BMI subgroups, univariate and multivariate logistic regression analysis of the relationship between uric acid and fundus arteriosclerosis in total populations.

Variable		Model 1[a]		Model 2[b]		Model 3[c]	
		OR, 95%CI	*p* value	OR, 95%CI	*p* value	OR, 95%CI	*p* value
Total							
BMI							
≤25	Q1	1.00(refer)		1.00(refer)	-	1.00(refer)	-
	Q2	1.71(1.41, 2.06)	<0.0001	1.09(0.87, 1.37)	0.46	1.04(0.83, 1.32)	0.72
	Q3	2.48(2.06, 2.99)	<0.0001	1.37(1.06, 1.75)	0.01	1.26(0.98, 1.63)	0.08
	Q4	3.20(2.64, 3.88)	<0.0001	1.98(1.50, 2.60)	<0.0001	1.76(1.32, 2.35)	0.0001
p for trend		<0.0001		<0.0001		<0.0001	
>25	Q1	1.00(refer)	-	1.00(refer)	-	1.00(refer)	-
	Q2	1.34(1.00, 1.79)	0.05	1.26(0.88, 1.81)	0.21	1.32(0.92, 1.90)	0.14
	Q3	1.06(0.81, 1.40)	0.66	1.01(0.71, 1.45)	0.95	1.05(0.73, 1.50)	0.81
	Q4	1.27(0.97, 1.66)	0.08	1.60(1.12, 2.29)	0.01	1.67(1.15, 2.41)	0.01
p for trend		0.30		0.0007		0.0010	

Notes: Cut-points: Total (Q1: ≤274.60, Q2: 274.61-339.20, Q3: 339.21-410.70, Q4: >410.70μmol/L or Q1: ≤4.61, Q2: 4.62-5.70, Q3: 5.71-6.90, Q4: >6.90mg/dL). BMI: body mass index; eGFR: estimated glomerular filtration rate; TG: triglycerides; TC: total cholesterol; LDL: low density lipoprotein; HDL: high density lipoprotein.

[a] Model 1: unadjusted. [b] Model 2: adjusted for age, gender, smoking and drinking.

[c] Model 3: adjusted for age, gender, smoking, drinking, eGFR, TG, TC, LDL and HDL.

Supplementary Table S3. In the age subgroups, univariate and multivariate logistic regression analysis of the relationship between uric acid and fundus arteriosclerosis in total populations.

Variable		Model 1[a]		Model 2[b]		Model 3[c]	
		OR, 95%CI	*p* value	OR, 95%CI	*p* value	OR, 95%CI	*p* value
Total							
Age							
≤50	Q1	1.00(refer)		1.00(refer)	-	1.00(refer)	-
	Q2	1.84(1.11, 3.04)	0.02	1.25(0.72, 2.15)	0.43	1.08(0.62, 1.87)	0.78
	Q3	2.48(1.53, 4.01)	0.0002	1.19(0.66, 2.14)	0.56	0.99(0.55, 1.78)	0.97
	Q4	5.35(3.46, 8.27)	<0.0001	1.68(0.92, 3.05)	0.09	1.23(0.67, 2.25)	0.51
p for trend		<0.0001		0.05		0.41	
>50	Q1	1.00(refer)	-	1.00(refer)	-	1.00(refer)	-
	Q2	1.42(1.20, 1.69)	<0.0001	1.28(1.07, 1.53)	0.01	1.23(1.03, 1.47)	0.02
	Q3	1.72(1.46, 2.03)	<0.0001	1.38(1.14, 1.66)	0.0009	1.29(1.07, 1.56)	0.01
	Q4	2.68(2.27, 3.15)	<0.0001	2.01(1.65, 2.44)	<0.0001	1.80(1.47, 2.21)	<0.0001
p for trend		<0.0001		<0.0001		<0.0001	

Notes: Cut-points: Total (Q1: ≤274.60, Q2: 274.61-339.20, Q3: 339.21-410.70, Q4: >410.70 μmol/L or Q1: ≤4.61, Q2: 4.62-5.70, Q3: 5.71-6.90, Q4: >6.90mg/dL). BMI: body mass index; eGFR: estimated glomerular filtration rate; TG: triglycerides; TC: total cholesterol; LDL: low density lipoprotein; HDL: high density lipoprotein.

[a] Model 1: unadjusted. [b] Model 2: adjusted for BMI, gender, smoking and drinking.

[c] Model 3: adjusted for BMI, gender, smoking, drinking, eGFR, TG, TC, LDL and HDL.

Supplementary Table S4. OR and 95% CI for changes in SUA for fundus arteriosclerosis incidence according to SUA as a continuous variable in total, males and females.

Variable	Model 1		Model 2	
	OR, 95%CI	*p* value	OR, 95%CI	*p* value
Total	1.004(1.004, 1.005)	<0.0001	1.002(1.001, 1.003)	<0.0001
Males	1.001(1.001, 1.002)	0.0002	1.002(1.001, 1.003)	<0.0001
Females	1.007(1.006, 1.008)	<0.0001	1.000(0.999, 1.002)	0.59

Notes: BMI: body mass index; eGFR: estimated glomerular filtration rate; TG: triglycerides; TC: total cholesterol; LDL: low density lipoprotein; HDL: high density lipoprotein.

Model 1: unadjusted.

Model 2: adjusted for age, gender, BMI, smoking, drinking, eGFR, TG, TC, LDL and HDL. Unadjusted gender variables in males and females.

Supplementary Table S5. Population distribution by clinical uric acid level in males and females.

Variable	Low (N, %)	Normal (N, %)	High (N, %)	Total
Males	54(0.43)	8228(65.17)	4343(34.40)	12625
Females	38(0.35)	9077(83.67)	1734(15.98)	10849
Total	92(0.39)	17305(73.72)	6077(25.89)	23474

Note: In males, the clinical reference value range of uric acid is 202.0-430.0 μmol/L or 3.4-7.2mg/dL. In females: 137.0-339.0 μmol/L or 2.3-5.7 mg/dL.

Uric acid is divided into three levels (low, normal, high level) according to clinical reference values.

Supplementary Table S6. OR and 95% CI for changes in SUA for fundus arteriosclerosis incidence according to SUA as a binary variable in males and females.

Variable		Model 1		Model 2	
		OR, 95%CI	*p* value	OR, 95%CI	*p* value
Males	Q1	1.00		1.00	
	Q2	1.33(1.19, 1.48)	<0.0001	1.42(1.23, 1.63)	<0.0001
Females	Q1	1.00		1.00	
	Q2	2.34(1.96, 2.80)	<0.0001	0.99(0.78, 1.27)	0.99

Notes: Cut-points: Males(Q1: <430.0μmol/L, Q2: ⩾430.0μmol/L or Q1: <7.2 mg/dL, Q2: ⩾ 7.2mg/dL); Females(Q1: <339.0μmol/L, Q2: ⩾339.0μmol/L or Q1: <5.7 mg/dL, Q2: ⩾ 5.7mg/dL). BMI: body mass index; eGFR: estimated glomerular filtration rate; TG: triglycerides; TC: total cholesterol; LDL: low density lipoprotein; HDL: high density lipoprotein.

Model 1: unadjusted.

Model 2: adjusted for age, BMI, smoking, drinking, eGFR, TG, TC, LDL and HDL.

Supplementary Table S7. OR and 95% CI for changes in hyperuricemia for fundus arteriosclerosis incidence according to whether it was hyperuricemia as a binary variable in males and females.

Variable		Model 1		Model 2	
		OR, 95%CI	*p* value	OR, 95%CI	*p* value
Males	No	1.00		1.00	
	Yes	1.31(1.18, 1.46)	<0.0001	1.39(1.21, 1.60)	<0.0001
Females	No	1.00		1.00	
	Yes	2.72(2.22, 3.32)	<0.0001	1.03(0.79, 1.36)	0.82

Notes: Cut-points: Males(without hyperuricemia or No: <420.0μmol/L, with hyperuricemia or Yes: ⩾420.0μmol/L or No: <7.1 mg/dL, Yes: ⩾7.1mg/dL); Females(without hyperuricemia or No: <360μmol/L, with hyperuricemia or Yes: ⩾360.0μmol/L or No: <6.1 mg/dL, Yes: ⩾6.1mg/dL).

BMI: body mass index; eGFR: estimated glomerular filtration rate; TG: triglycerides; TC: total cholesterol; LDL: low density lipoprotein; HDL: high density lipoprotein.

Model 1: unadjusted.

Model 2: adjusted for age, BMI, smoking, drinking, eGFR, TG, TC, LDL and HDL.

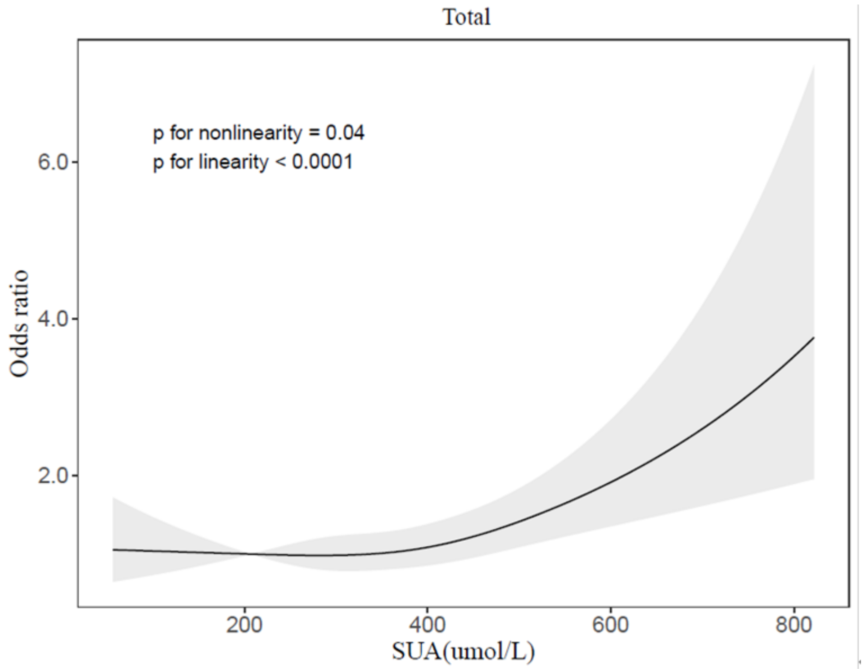

Supplementary Figure S1. Association of SUA with the incidence of fundus arteriosclerosis according to restricted cubic spline regressions using four knots in total populations (percentiles 5, 35, 65 and 95), with the reference point set at percentile 12.5. Odds ratios were adjusted for age, gender, BMI, smoking, drinking, eGFR, TG, TC, LDL and HDL.

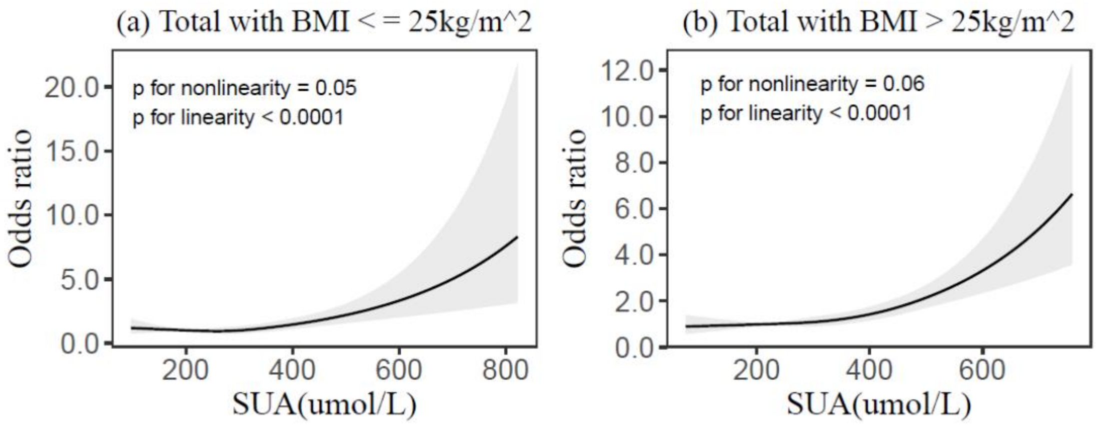

Supplementary Figure S2. In the BMI subgroups, association of SUA with the incidence of fundus arteriosclerosis according to restricted cubic spline regressions using four knots in total populations (percentiles 5, 35, 65 and 95), with the reference point set at percentile 12.5. (a) (b) Odds ratios were adjusted for age, gender, smoking, drinking, eGFR, TG, TC, LDL and HDL, respectively.

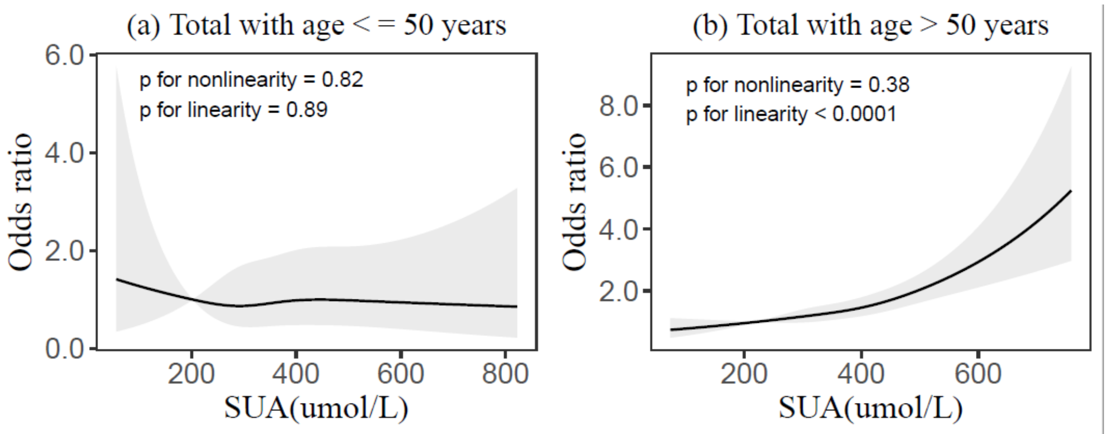

Supplementary Figure S3. In the age subgroups, association of SUA with the incidence of fundus arteriosclerosis according to restricted cubic spline regressions using four knots in total populations (percentiles 5, 35, 65 and 95), with the reference point set at percentile 12.5. (a) (b) Odds ratios were adjusted for BMI, gender, smoking, drinking, eGFR, TG, TC, LDL and HDL, respectively.
